# How to Improve Integrated Care for People with Chronic Conditions: Key Findings from EU FP-7 Project INTEGRATE and Beyond

**DOI:** 10.5334/ijic.3096

**Published:** 2017-09-25

**Authors:** Liesbeth Borgermans, Yannick Marchal, Loraine Busetto, Jorid Kalseth, Frida Kasteng, Kadri Suija, Marje Oona, Olena Tigova, Magda Rösenmuller, Dirk Devroey

**Affiliations:** 1 Faculty of Medicine and Pharmacy, Department of Family Medicine and Chronic Care, Vrije Universiteit Brussel, Brussels, BE; 2 Tranzo Scientific Center for Care and Welfare, Tilburg University, Tilburg, NL; 3 SINTEF Technology and Society, Health Services Research, Trondheim, NO; 4 Department of Family Medicine, Institute of Family Medicine and Public Health, Faculty of Medicine, University of Tartu, Tartu, EE; 5 Center for Research in Healthcare Innovation Management, IESE Business School, ES

**Keywords:** integrated care, policies, chronic care

## Abstract

**Background:**

Political and public health leaders increasingly recognize the need to take urgent action to address the problem of chronic diseases and multi-morbidity. European countries are facing unprecedented demand to find new ways to deliver care to improve patient-centredness and personalization, and to avoid unnecessary time in hospitals. People-centred and integrated care has become a central part of policy initiatives to improve the access, quality, continuity, effectiveness and sustainability of healthcare systems and are thus preconditions for the economic sustainability of the EU health and social care systems.

**Purpose:**

This study presents an overview of lessons learned and critical success factors to policy making on integrated care based on findings from the EU FP-7 Project Integrate, a literature review, other EU projects with relevance to this study, a number of best practices on integrated care and our own experiences with research and policy making in integrated care at the national and international level.

**Results:**

Seven lessons learned and critical success factors to policy making on integrated care were identified.

**Conclusion:**

The lessons learned and critical success factors to policy making on integrated care show that a comprehensive systems perspective should guide the development of integrated care towards better health practices, education, research and policy.

## Introduction

The ageing population and the increase in the number of people diagnosed with chronic conditions are forcing policy makers and public health leaders to reform healthcare systems at an increasing speed [1,2,3,4]. In the EU 27, population over 80 years will grow from 5% in 2010 to 11,5% in 2050 [5]. Between 20–40% of patients aged 65 and over are having multi-morbidity, characterized by more than five chronic conditions [6]. The Global Burden of Disease Study 2013 (GBD 2013) has shown the number of Disability Adjusted Life Years (DALYs) increased for most non-communicable diseases between 2005 to 2013 [7]. Multimorbidity is strongly associated with higher mortality, poorer quality of life and functional status, and higher rates of health service use including emergency hospital admission [8,9]. In the EU 27, the amount of money spent on medical care is increasing faster than the gross domestic product (GDP) in most countries [10]. However, a constant growth of services might not be affordable, nor will the labour market support ever continuing expansion [11]. In addition, the financial and economic crisis and the introduction of austerity measures in many EU countries, contribute to a renewed context for health care policies directed at people with chronic conditions [12].

All these drivers for change are now necessitating significant change, and policy makers have a key role to play in enabling successful progress [13]. Any policymaker that aims for the Triple Aim (guaranteeing the equitable provision of high-quality, evidence-based care at a reasonable cost), should acknowledge that health challenges cannot be confronted successfully by actors working in isolation nor by reductionist approaches that suggest a limited set of interventions (e.g. financial incentives) to improve the care for people with chronic conditions [14,15]. Integration assembles diverse actors and organisations in a collective effort to design and deliver new service models underpinned by multidisciplinary working and generic practice [16]. Integrated management of noncommunicable diseases makes sense for at least three important reasons [17,18]. First, since most people have more than one risk factor and/or chronic condition/illness (e.g. hypertension and obesity) [19], it makes sense to treat their conditions within an integrated framework of care. Second, most chronic diseases place similar demands on health workers and health systems, and comparable ways of organizing care and managing these conditions are similarly effective regardless aetiology [19]. Third, most chronic diseases have common primary and secondary risk factors. In addition to integrated management of chronic diseases, general integration of this type of care within health services is essential. Chronic disease should not be considered in isolation but rather as one part of the health status of the individual. Health in this context means ‘the ability to adapt and to self-manage in the face of social, physical, and emotional challenges’ [20].

The defining questions for the future are not whether integrative or collaborative practices have the intended effect. Rather, the central question is how we can change health care systems to achieve the best outcomes. In doing so we should use evidence-based truths to build a framework for changing the national and international policy narratives about integrated care. In the new paradigm, the patient, not the health professionals would be at the centre of the universe. That shift will have enormous system-wide consequences, since the interests supporting the status quo are formidable, and the complexity of the change process substantial [21]. Leading in complexity requires leaders to accept the complexity, create an adaptive space in which innovation and creativity can flourish and then integrate the best practices that emerge into the formal organizational structure [22].

With this study, we put forward the lessons learned and critical success factors to policy making on integrated care, as identified from the EU FP-7 Project INTEGRATE (www.projectintegrate.eu) and a number of other sources. Project Integrate aimed to gain insights into the leadership, management and delivery of integrated care to support European care systems to respond to the challenges of ageing populations and the rise of people living with long-term conditions. The project was conducted over a four-year period (2012–2016) and included partners from nine European countries.

This paper is the first in a series of papers on how to improve integrated care for people with chronic conditions.

## Methods

Lessons learned and critical success factors to policy making on integrated care were identified through consultation of five different sources (Table 1). The first source were findings and recommendations from the different work packages of the EU Project INTEGRATE. A second source was a literature review on integrated care policies for people with chronic conditions (available upon request). Four additional sources used were a) existing frameworks on chronic and people-centred/integrated care, b) key findings from other EU Projects targeting chronic illnesses/integrated care and c) a selected set of ‘best practices’ on integrated care from different countries and d) our own experiences with research and policy making in integrated care at the national and international level [23,24,25,26,27,28,29,30].

**Table 1:** Overview of sources to the identification of policies on integrated care.

Sources	Content

**1. Project INTEGRATE Work Packages**	Work Package 2: Case study COPD Work Package 3: Case study diabetes Work Package 4: Case study geriatric conditions Work Package 5: Case study mental conditions Work Package 6: Care Process Design Work Package 7: HR management/skill mix Work Package 8: financial flows & barriers Work Package 9: patient involvement Work Package 10: IT -management Work Package 11: International Check Work Package 12: Practical Managerial Lessons
**2. Literature review on policies for people with chronic conditions**	A full overview of the research methodology and findings is available upon request
**3. Existing Frameworks on chronic and integrated care**	– Chronic Care Model [31] – The Innovative Care for Chronic Conditions (ICCC) Framework [32] – The WHO European Framework for Action on Integrated Health Services Delivery [33] – The WHO Global Strategy on People-centred and Integrated Health Services [23] – The Rainbow Model of Integrated Care (RMIC) [34]
**4. EU Projects/initiatives targeting ageing/chronic illnesses and/or integrated care**	– **The European Innovation Partnership on Active and Healthy Ageing** – The **Age Platform** – **FUTURAGE** (to create a roadmap for future research into the issues of ageing within society – **BRAID** (Bridging Research in Ageing and ICT Development – The Joint Action on Chronic Diseases **(JA-CHRODIS)** that addresses chronic diseases and promoting healthy ageing across the life cycle – ‘Empowering Patients in the management of chronic diseases’ **(EMPATHiE)** project, which aims to achieve a common understanding of the concept of patient empowerment and identify good practices, success factors and barriers – The **EU-WISE** project ‘Self-care for Long-Term Conditions in Europe’ under the 7th Framework Programme of the European Commission which aimed to understand the role and influences of resources external to health services which have an impact on people’s capacities to manage long-term conditions – Sustainable tailored integrated care for older people in Europe **(SUSTAIN)** – OPtimising thERapy to prevent Avoidable hospital admissions in the Multimorbid elderly **(OPERAM)** under the H2020 programme of the European Commission – The Active Ageing with Type 2 Diabetes as Model for the Development and Implementation of Innovative Chronic Care Management in Europe **(“MANAGE-CARE”)** analyses Chronic Care Programs paying special attention to components which are effective, problematic and missing – The Promoting personalized and patient-centred healthcare **(PERSPeCtive)** project focuses on the development of patient-oriented primary care for chronic diseases in the ageing population.
**5. Best practices on patient-centered and integrated care**	– Compendium of initiatives in the WHO European Region (Lessons from transforming health services delivery), 2016. [35] – Synthesis of case studies on patient-centered and integrated care from OECD health systems, 2015. [12] – European Innovation Partnership on Active and Healthy Ageing (2012). Replicating and tutoring integrated care for chronic diseases, including remote monitoring at regional levels. Brussels: B3 Action Group. [36] – RAND Europe (2012). National evaluation of the department of health’s integrated care pilots. Cambridge: RAND Corporation. [37] – Accountable Care Organizations (ACOs) (US) [38,39] – Health Maintenance Organizations (HMOs) (US): Kaiser Permanente, Marshfield Clinic, Carle Clinic, Geisinger Health System [40] – Managed Clinical Networks and chains of care (Scotland, Sweden) [41] – Disease Management Programmes (The Netherlands, United Kingdom) [42]

## Results

### The lessons learned

Seven major lessons have been identified that can be summarized as: 1) ‘it is about compassionate and competent care’, 2) ‘it is about disruptive innovation’, 3) ‘it is about competencies’, 4) ‘it is about the broader picture of well-being’, 5) ‘it is about effective implementation strategies’ 6) ‘it is about context’, 7) ‘it is about outcomes’.

#### Lesson 1: “It is about compassionate and competent care”

Many of the current chronic illness care strategies have emanated from the Wagner Chronic Care model (CCM) [43] and the Innovative Care for Chronic Conditions (ICCC) Framework [44]. Different interpretations of the CCM and ICCC have led to the proliferation of disparate integrated care programmes/interventions within the chronic illness spectrum as a whole [27,28,45,46,47,48,49,50]. The increasing complexity attributed to the concept of integrated care, from a theoretical, operational and implementation perspective has resulted in growing confusion over its meaning and outcomes. The constructs commonly described in scoping literature include patient-centered care, care coordination, continuity of care, chronic disease management, integrated healthcare delivery, amongst others [17,18,25,29,34,41,51,52,53,54,55,56,57,58,59,60,61,62]. Findings from Project Integrate have shown there is an increasing need to speak with one voice when talking about integrated care. The different case studies as they were developed in the context of Project Integrate essentially reflect many of the components of what is considered ‘compassionate and competent care’. The latter type of care is essentially integrated, people-centered and values a bio-psycho-social approach to care emphasizing the importance of equity, and high-quality interventions across the life course and the entire health continuum and aims at better care experiences, health outcomes, and with a more efficient use of resources.

What is essential to compassionate and competent care is that it focuses on those aspects of care that are directly and intrinsically important to people, rather than the inputs and outputs that might be used to deliver those outcomes. The question to patients: ‘What matters the most to you’ should drive how ‘compassionate and competent care’ is operationalized. It thus focuses on outcomes that are both objective and intrinsically subjective, recognizing that objective evidence about people’s life circumstances can be usefully complemented by information about how people experience their lives. It also considers the distribution of chronic care outcomes across the population as an important feature to reflect in measurement, including disparities associated with age, gender, education and income [20]. This notion of ‘population health’ is essential to compassionate and competent care. It is defined as ‘the health outcomes of a group of individuals’ including the distribution of such outcomes within the group [63]. There are three competing models for producing health and improving health in a population [64] including the medical, the public health and the social determinants of health model. All three models must be pursued in balance.

#### Lesson 2: “It is about disruptive innovation”

Project Integrate has shown that policy makers need to consider ‘disruptive innovation’ when designing policies that target improvements in care for people with chronic conditions. This type of innovation does not exclude the use of a stepwise approach to change. There are two basic approaches to developing such health policies. The first, which is cautious and careful (a small idea and a small intervention or even a big idea and a small intervention), is more likely to be tested and implemented because institutions and professionals will not be threatened by the magnitude of the change. But this approach runs the risk of discrediting the concept that is being tested because what is being implemented is too limited, circumscribed, or piecemeal [64]. Making marginal change runs the risk of wasting time, and the crisis facing European health care systems requires more than marginal change. The second approach is disruptive and daring (big idea and big intervention) [64]. Disruptive Innovation is a type of innovation that creates new networks and players and tends to displace existing structures and actors, and is as such a real paradigm shift. Achieving value and controlling costs will require disruption regarding how care is delivered and how we reward people for producing services.

Project Integrate has shown that policy makers need to opt for comprehensive disruptive change, not innovation at the margins. Disruptive innovation does not counteract the use of a stepwise approach. But the magnitude of the change required is so great that it is not enough to address health policies in a sequential manner, nor is it sufficient to (only) apply top-down strategies at the organisational level (e.g. funding, governance, accountability) [65]. There is however no “one-size-fits-all” solution for monitoring, managing and stimulating the adoption of disruptive innovations [66]. The areas of main focus for disruptive innovations in health care are new models and interventiions of person-centred community-based health delivery that allow a decentralisation from traditional health care venues, such as hospitals, to integrated care models (e.g. mobile multidisciplinary teams providing mental health at home). Other areas of disruptive innovation are new technologies that allow early diagnostics and personalised medicine, promotion, community-based therapy and care and the empowerment of patients/citizens, as well as potential curative technologies (e.g. regenerative medicine, immunotherapy for cancer). A last set of examples of disruptive innovations are person-oriented approaches for the treatment of patients with multiple chronic diseases, situations of frailty and/or of loss of functionalities in a multi-cultural context, education of the health workforce and transfer of skills and tasks from highly trained, high cost personnel to personnel that have less specialised trained and are more affordable (e.g. from generalists to nurses, and ultimately to patients themselves) It is important to note that large-scale disruptive innovation might be frightening, and needs clear and convincing risk identification and control. Some people (including patients and carers with new responsibilities) might perceive loss of function, control, income and status and will probably oppose the innovation. There is a need to address these challenges openly by policy makers to make the ‘multi-stakeholder simultaneous parachute jump work’, as demonstrated by the Project Integrate case studies on mental health, geriatric care, diabetes and COPD.

The implementation of a disruptive innovation requires the creation of new organisational models and management plans, the presence of favourable framework conditions, and the development of new models of commissioning and financing (incentives for its adoption and diffusion) [66]. Adoption and diffusion of any disruptive innovation should always be based on evidence deriving from a specific in-depth evaluation that takes into consideration elements such as the potential costs and benefits of the disruptive innovation, the potential costs and benefits of transformation, the reversibility of choices, the type of barriers to be overcome, and the aspects of uncertainty [66].

#### Lesson 3: “It is about competencies”

Project Integrate has shown the successful development of integrated care requires new types of competencies. The process of matching health workforce competencies to patient needs involves more than just securing a health workforce that has the theoretical knowledge and skills to work more efficiently and effectively [67,68]. Competency clusters for integrated health services include governance, patient advocacy, effective communication, team work, people-centered care, quality assurance and the willingness for continuous learning [68]. The need to prepare the health workforce for this paradigm shift is urgent [67]. Especially the health professionals of the future will need to partner with the patient in facilitating care and maintaining health. When health professionals partner with patients and families, patients make more informed choices about their care, use medications more safely, practice more effective self-management, contribute to infection-control initiatives, and help reduce medical errors—all translating into measurable improvements in the quality and safety of care [69]. Patients and their families can also be expected to master competencies for integrated care. In particular, patient’s competencies include making informed decisions, playing an active role in defining their care plan, complying with agreed upon treatments and, overall, taking responsibility for their own health and wellbeing [68]. Self-care is defined as: *“What individuals, families and communities do with the intention to promote, maintain, or restore health and to cope with illness and disability with or without the support of health professionals such as pharmacists, doctors, dentists and nurses”*. It includes but is not limited to self-prevention, self-diagnosis, self-medication and self-management of illness and disability” [70]. This assumes mental competence, health literacy and supported decision-making – factors that are still grossly under-addressed in Europe. Also, leadership competencies are required to bring about the fundamental changes we need [71,72]. Leadership takes many forms and varies importantly according to task and context [73]. Leadership is defined as ‘*the perception or acceptance by members of a group of their superior’s ability to inspire, influence and motivate them to meet their goals and contribute to the achievement of shared objectives*’ [74]. Traditional hierarchical ‘concentrated’ leadership is associated with particular positions, while distributed leadership involves those with particular skills and abilities across multiple levels [75]. Strategic level stakeholders see the most effective form of leadership for integrated care as one that blends distributed and concentrated leadership [71,76]. Components of effective leadership are: building transformational relationships, defining collaboratively oriented values, supporting the development of shared meanings about change, instilling a culture of collective inquiry and mutual accountability, role-model management practices, effective communication and flexibility, engagement with patients and families, care coordination support, and staff development, amongst others.

#### Lesson 4: “It is about broader picture of well-being”

Project Integrate has shown that in order to meaningfully improve the care for people with chronic conditions it is paramount to take into account the broader determinants and thus the ‘big picture’ of well-being. Whilst health services themselves are important for health, they are not the only relevant services - essential to good health is good nutrition, domestic and personal hygiene, access to technical aids, safe housing, and socialisation [11]. Sustainable and equitable improvements in health and well-being in people with chronic conditions consequently are the product of effective policy across all parts of government and collaborative efforts across all parts of society [77]. While there is no single recipe for well-being, there is an increasing consensus around a common list of useful ingredients. The OECD Framework for measuring individual well-being [78] includes eleven different dimensions that are important for well-being today grouped under the two broad headings: *material conditions* (income and wealth, jobs and earnings, housing), and *quality of life* (health status, work-life balance, education and skills, social connections, civic engagement and governance, environmental quality, personal security and subjective well-being). Well-being is thus experienced at the subjective, individual level and it can also be described objectively through several indicators at the population level. Engaging with the full complexity of subjective well-being demands a multidisciplinary, integrated health approach, ie. an eco-bio-psycho-social approach to care.

#### Lesson 5: “It is about effective implementation strategies”

Project Integrate has shown that seemingly good ideas to promote integrated care do not always result in changes in practice. The use of centralised top-down strategies including e.g. contractual arrangements and regulatory frameworks often fail to demonstrate improved outcomes [79]. Integrated structures are not enough in themselves to secure integrated service delivery, nor does the form of integration necessarily affect the effectiveness of the service [71]. Integrated structures without enabling implementation strategies may therefore not translate into performance improvement [80]. For this reason, it is important to understand how new ways of working are introduced, sustained and become established in day-to-day practice [81]. The question of sustainability is crucial if the gains in patient care that derive from innovations are to be maintained, rather than lost to an ‘improvement-evaporation effect’ [82]. Implementation strategies in this sense act as the barriers or facilitators to any integrated care programme. Examples of evidence-based implementation strategies are: a shared mission and vision (shared ambition), shared values, sense of urgency, instrumental and transformative partnerships, an understanding of different roles and responsibilities within a team, training to reflect changing roles and responsibilities, communication, attitudes, organizational culture, IT-systems, the use of quality-management systems linking plan-do-study-act cycles at different levels, funding arrangements, governance arrangements among partners, learning organisations, training and career progression, change management, the use of quality norms based on realised successes, performance agreements with multiple stakeholders, organisational support, monitoring, and quarterly accountability reports [83,84]. Effective integration strategies are often linked to social relationships in which people interactively assign, re-interpret and re-negotiate their identities, values and working methods [41,54].

#### Lesson 6: “It is about context”

Experiences from Project Integrate have shown that any viable health policy needs to be compatible with the nation’s value system as it applies at the local, regional and national level. In this context, discussion on the respective roles of national policy makers and local units of government is essential. Within countries there are differing socioeconomic, cultural, geographical, political and health system realities that provide the context that must inform the way integrated care is adopted [85]. Integrated care is in this sense a complex, interdisciplinary, nonlinear and dynamic change process [86]. Integrated care programmes are developed in very different contexts with unique characteristics and dynamics and it is especially the local context that matters the most [14,87]. The notion of ‘complex adaptive systems’ applies to integrated care as such systems have the tendency to learn, adapt and self-organise in response to continuous feedback from changing patterns of relationships and interactions among all stakeholders and the environment in which they operate [88].

#### Lesson 7: “It is about outcomes”

Findings from Project Integrate have shown that many countries struggle with what to measure related to the execution of patient-centered and integrated care. A number of countries have developed a set of quality indicators that accompany the introduction of integrated health services. A well-known example comes from the UK where a range of generic indicators for measuring the quality of integrated care has been developed (i.e. 35 indicators across six key domains of quality) [89]. Especially ‘Triple Aim’ indicators play an important role in policy formulation and comparison of the effectiveness of patient-centred and integrated care interventions [90,91]. Project Integrate has shown that even though measuring the impact (outcomes) of integrated care interventions is important, systematic collection of evidence is nowadays not in place in this field [92,93,94,95,96,97]. Instead, integrated care measurement largely focuses on measuring individual aspects of integrated care [96]. Current measures are more aspirational than an integral yardstick of society. There is a need for comprehensive instruments to measure integrated care that reflect the comprehensive nature of the concept of integrated care at the structure, process and outcome level of care.

### Key success factors

Seven key success factors to policy making on integrated care were identified, including political leadership, the use of a unifying framework, stepwise approach and a clear scaling strategy, the need to establish inter-sectoral action, instrumental and transformative partnerships, and the development of an evidence-based narrative on integrated care.

#### Key success factor 1: Demonstrate political and clinical leadership

The Minister of Health, regional and local health administrators and health professionals have a role as both an advocate for patients, the interests of health facilities and health workers and as the agency responsible for ensuring that government health system objectives are met [98]. Political leadership is characterized by multiple features [99,100,101], and stewardship is intended as the capacity of the Ministry of Health to initiate and lead the necessary interventions and to overcome “system inertia” [102]. Numerous policy papers and academic contributions across a range of countries also emphasize the importance of clinical leadership in chronic care reform [103]. Clinical (physician) leadership may play a role in stimulating quality improvement and new innovations in service design, with positive consequences for patient safety and satisfaction. The system’s problems should not be addressed only by politicians, who are virtually powerless to effect meaningful change in health care until physicians fix the way care is delivered [64]. Physicians must become a constructive voice in deciding how costs attributed to integrated and chronic care can more appropriately reflect society’s values and needs. Planning for that eventuality should begin now, but cannot be led by a single specialty organization, cannot aggravate the town/gown split in medicine, cannot conclude by protecting the salaries of physicians relative to the salaries of other health care professionals, and cannot be performed in a way that violates the Hippocratic oath [64].

Important tools for creating transformative partnerships are Community Health Applied Research Networks, Chronic Illness Research Centers, and Health Boards, amongst others.

#### Key success factor 2: Use a unifying framework

It is essential for policy makers to make use of a unifying framework for integrated care to ensure that actions at all levels and by all sectors are mutually supportive. Several organisational models for integrated care have been proposed and implemented internationally. Perhaps the best known and most influential is the Chronic Care Model that has been adopted or adapted by many countries. Recent important frameworks include the WHO Framework for Action towards Coordinated/Integrated Health Services Delivery (CIHSD) [33] and the WHO global strategy on people-centered and integrated health services [104].

#### Key success factor 3: Use a stepwise approach

Although health policies vary greatly in cost it will inevitably be easier for wealthy countries than poor ones to introduce many policies, especially those based on service provision. But some variations reflect differences in available resources, while others reflect differences in willingness to take action, as illustrated by the fact that neighbouring countries in similar economic conditions sometimes have very different outcomes. The European experience suggests that, in general, chronic care policies tend to follow national income, but in some cases, governments seem to be in the lead, doing more than might be expected, while in others they lag behind, doing less. Overall, it seems important to make use of a stepwise approach, particularly in countries that do not have sufficient resources to carry out all recommended actions.

#### Key success factor 4: Use a clear scaling strategy

There are different ways of thinking about scaling up integrated care model and programmes [105]. One approach is to simply enlarge the models to cover a wider catchment area or population. However, this would mean increasing the number of partners to ensure adequate service delivery for a larger population [106], which can be challenging. Another way of thinking about scaling up is to copy the successful model and implement elsewhere and so sustain local identity. While this appears feasible in some settings, it raises questions of implementability in areas with a different socio- economic and demographic context and different providers [107].

#### Key success factor 5: Establish inter-sectoral action (HiAP)

It is important for policy makers to develop multi-sectoral policies and partnerships for the development of integrated care targeting chronic disease prevention and control [19]. Health in All Policies (HiAP) promises to improve population health by harnessing the energies and activities of various sectors [108]. Non-health areas of public policy such as fiscal policy, social protection, education, transport and regional development (among others) can have an important effect on access to health services [66], and are essential to any effective strategy in response to non-communicable diseases [108].

#### Key success factor 6: Create instrumental and transformative partnerships

It is important for policy makers to create instrumental and transformative partnerships with patients and their families, civil society, professional caregivers, the private sector, universities and international organizations.

Especially the involvement of patients and Civil Society Organisations in policy making on integrated care is essential [109]. This will allow to eliciting patients’ views, not only on ‘what works’ for patients but also on the need for intervention and on factors influencing the implementation of particular health technologies, their appropriateness and acceptability [110]. Policy makers often fail to involve the very people who use healthcare services: patients, their families and community members [111]. Recent European health strategies and programmes declare service user involvement to be essential in the development and evaluation of policy and services [112]. It is agreed that feedback from patients and their families should be more rigorous and used to inform practice, not merely collated for research.

#### Key success factor 7: Develop an evidence-based model for chronic care evaluation

In order to develop an evidence-based model for chronic care evaluation it is important for policy makers to strengthen country capacity for surveillance and research on chronic diseases, their risk factors, and their determinants and to utilize the results of this research to support evidence-based policy and programme development [113]. National governments need to be ambitious in measuring progress towards delivery of integrated care that will address the prevention and management of chronic illnesses [114]. Most international policy frameworks have come forward with indicators that directly and indirectly allow measuring progress against pre-defined targets for chronic diseases and/or integrated care [23]. In this context, it is important to note that future research on integrated care for chronic diseases will increasingly rely on better electronic communication to coordinate care (based on shared client and professional views), and ‘in vivo’ quality measures. The integration of large datasets will become increasingly important, which range from electronic health records, over population and patient cohorts and registries and data on lifestyle, socioeconomic status, and so forth. Efficient use of ‘big data’ requires interoperability and stardardisation of different datasets, and requires public acceptance based on assurance of the protection of the privacy of individuals. In this context, partnerships between higher education institutions and local health services are needed to increase capacity and capability to produce and implement research through sustained interactions between academics and health services [115,116].

## Conclusion

Based on the findings from Project Integrate and other sources we argue that a comprehensive systems perspective should guide the development of integrated care towards better health practices, education, research and policy. Both the seven lessons learned and critical success factors discussed are considered essential to the development of this comprehensive systems perspective and effective implementation in a EU context and beyond. We consider our findings equally important to health care systems that apply a Bismarck or Beveridge model or a national health insurance model.
